# Supplementary material for: Time-resolved single dopant charge dynamics in silicon
Source: Nat Commun. 2016 Oct 26;7:13258. doi: 10.1038/ncomms13258 (PMC5095180; doi:10.1038/ncomms13258)
Supplement: Supplementary Information — Supplementary Figures 1-6, Supplementary Notes 1-2 and Supplementary References [file ncomms13258-s1.pdf]

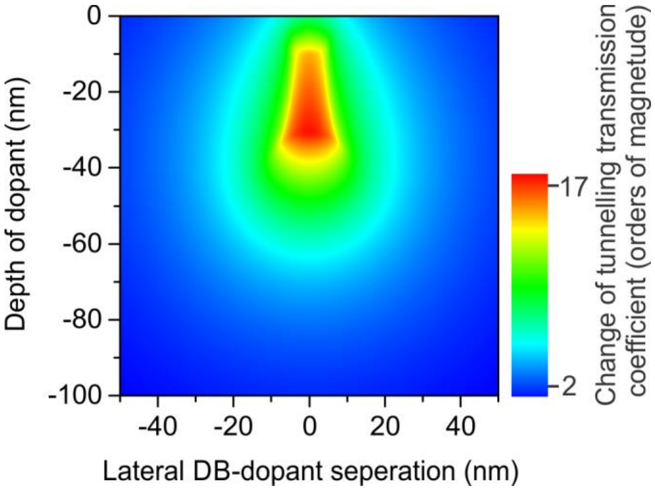

Supplementary Figure 1 | Change of the Tunnelling Transmission Coefficient from the Bulk to the Surface as a result of dopant ionization

Colour-map of change of the tunnelling transmission coefficient through the energy barrier between the degenerately doped bulk CB and the surface resulting from dopant ionization at different depth, and lateral position from a DB. The weights were calculated using WKB approximation. The sample bias voltage is set to -2.0 V. The tip and DB are placed at (0,1 nm) and (0,0), respectively.

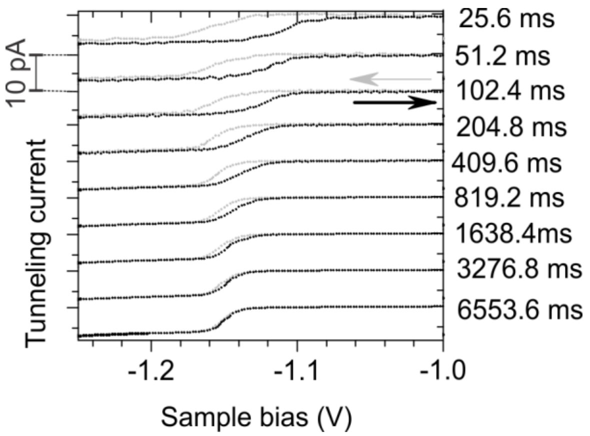

Supplementary Figure 2 | Additional Example: Time-resolved STM on a slow switching DB (DB2)

Forward (-1.2 V to -1.0 V) and reverse bias spectroscopy sweeps in the case of a DB with slow time dynamics reveals conductance hysteresis. The *I(V)* curves are the averages of 100 sweeps at different total sweep times indicated on the side of each curve. The hysteresis loop closes for sweep times around 6.5 s, indicating dynamics in the range of hertz at the transition bias.

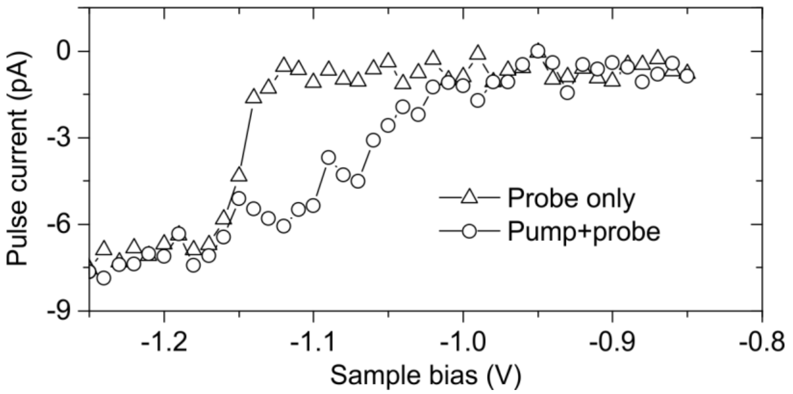

Supplementary Figure 3 | Time resolved *I*(*V*) spectroscopy on the DB in supplementary Fig. 2.

Current from a 1 μs width probe pulse with and without a 10 μs width preceding pump pulse. The delay from the trailing edge of the pump pulse to the leading edge of the probe pulse is 10 ns and the DC bias voltage is set at -0.80 V. The amplitude of the pump pulse is 0.60 V and that of the probe pulse is swept from 50 to 450 mV. Since the polarity of both pulses is negative, the pumped bias is -1.40 V and the probe sweeps the bias range of -0.85 to -1.25 V. The time-resolved *I*(*V*) measurement shows a wider hysteresis loop than any of the conventional STS sweeps, in agreement with the trend of widening of the hysteresis loop for faster *I(V)* sweeps observed in Supplementary Fig. 2. The time-resolved spectra measured with and without the pump pulse are analogous to forward and reverse sweeps of the conventional spectra shown in Supplementary Fig. 2, respectively.

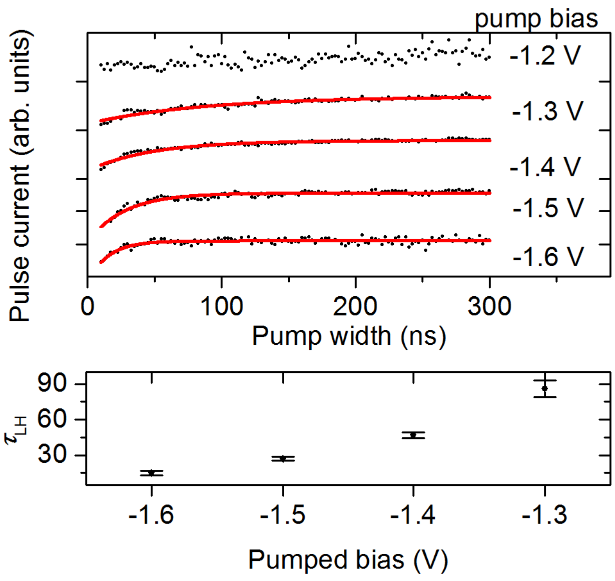

Supplementary Figure 4 | Time-resolved STM measurement of τ_{LH} at different pump bias on the DB in Supplementary Fig. 2.

The DC offset is -0.80 V. The probe width and relative delay is 1 μs and 10 ns, respectively. Red curves are exponential fits. (b) τ_{LH} versus pumped bias. Error bars indicate the standard errors of the exponential fits.

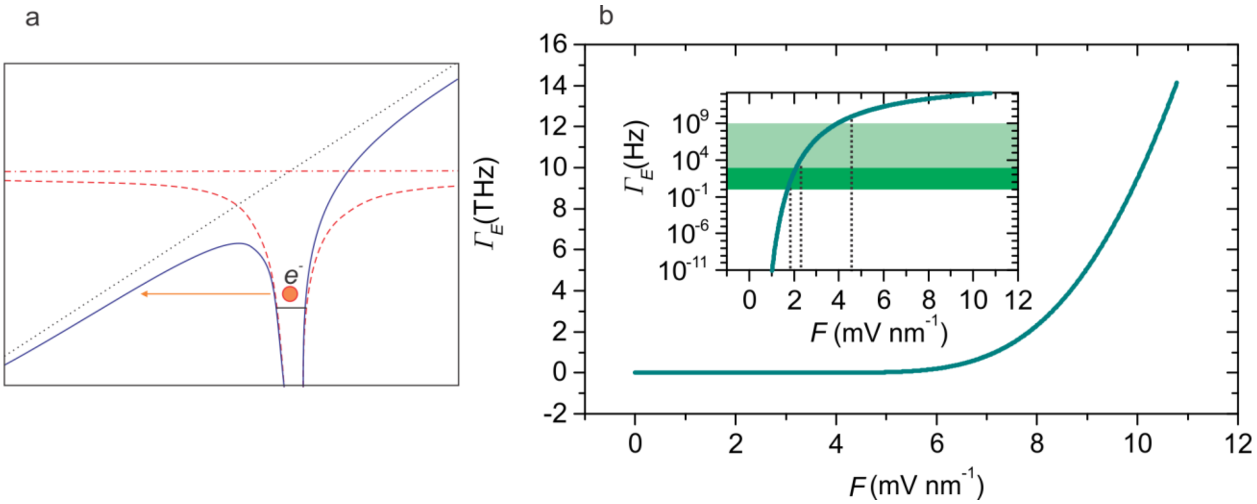

Supplementary Figure 5 |

(a) The arsenic dopant can be stripped of its bound electron through tunnel ionization in the presence of a field. (b) Ionization rate as a function of field strength. Inset shows a semi-logarithmic plot with the dark shaded region indicating the typical timescales for standard STM measurements. The lighter shaded region indicates the timescales that are accessible in time-resolved STM.

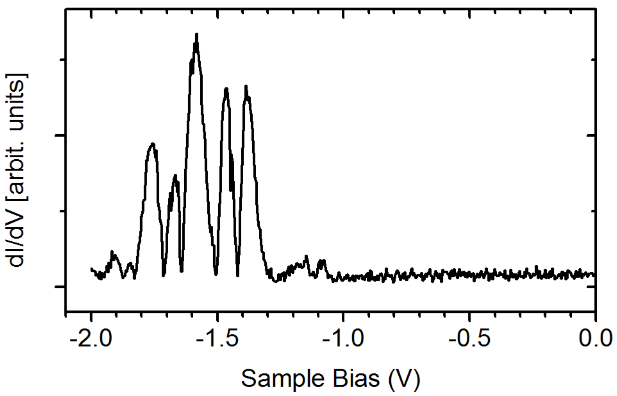

Supplementary Figure 6 | Multiple dopant ionization signatures in filled state tunnelling spectroscopy of a DB

DC tunnelling spectroscopy of DBs on samples prepared at 1250°C exhibit a wide range of critical voltages and signatures of multiple dopant ionizations. Here, we show an example with several features indicative of dopant ionization events.

Supplementary Note 1 | Signal Pair Analysis of Slow Transitions in the Bistable Energy Regime

The transition rates between the high conductance and low conductance states were calculated using a form of signal-pair analysis[1]. The current-traces were placed in histograms and the high conductance and low conductance states were fit with Gaussian functions. Regions of the histograms within each state were selected where the contributions from other states were minimized. Correlations were then calculated between these selected regions based on a linear transition pathway. The transition rates were determined directly from the fit of derived correlation functions[1] to these generated correlation data.

Supplementary Note 2 | Theoretical Treatment of Tunnel Ionization of Neutral As Dopants

Dopants are ionized in the field of the tip. The bound electron tunnels through a roughly triangular barrier formed by the confining potential of the host dopants and the tilted potential of the field, as shown in Supplementary Fig. 5a. Ionization rate is a function of field strength, which can be derived analytically for the Hydrogen atom,

$$\Gamma_{\text{ion}}\left(F\right)=\frac{4m^3e^9}{\hbar^7F}\exp\left[\frac{-2m^2e^5}{3\hbar^4F}\right]. \tag{1}$$

To treat the case of an arsenic dopants in silicon, we adapt this relation and express it in terms of the binding energy, as

$$\Gamma_{\text{ion}}\left(F\right)=\frac{128\pi\varepsilon E_{\text{bind}}}{\hbar e^3F}\exp\left[\frac{-32\pi\varepsilon E_{\text{bind}}}{3e^3F}\right], \tag{2}$$

which is the inverse of the time constant, τ_{LH}, in equation (1) of the manuscript. The binding energy and dielectric constant in this equation are modified from the case of the hydrogen atom in vacuum to reflect the binding energy of the arsenic dopants and the dielectric constant of silicon. This form of the ionization rate as a function of field strength does not explicitly include the effective mass of the electron, instead using the known binding energy of 54 meV. This is done since the effective mass treatment of an electron dopants in silicon underestimates the binding energy by a factor of nearly two. Since the binding energy plays a very important role in determining the rate of tunnel ionization, we choose to insist on this quantity in our treatment of dopants.

Supplementary Fig. 5b shows ionization as a function of field strength. The rate increases non-linearly, and within the fields that can be experienced by near-surface dopants, we see that there is a dramatic change in the ionization rate from effectively zero, to much faster than the timescales that are accessible in STM experiments, shown as shaded regions in the inset of Supplementary Fig. 5b. The sharp transition studied in this paper is due to this rapid transition in rates as the field experienced by individual dopants increases.

Supplementary References

1. Hoffmann, A. & Woodside, M. T. Signal-Pair Correlation Analysis of Single-Molecule Trajectories. *Angew. Chemie Int. Ed.* **50,** 12643–12646 (2011).
